# ASSOCIATION BETWEEN BRAIN VOLUMES AND PATTERNS OF COMMUNITY-DWELLING PHYSICAL ACTIVITY

**DOI:** 10.1093/geroni/igz038.1498

**Published:** 2019-11-08

**Authors:** Amal A Wanigatunga, Yang An, Christos Davatzikos, Jacek K Urbanek, Adam P Spira, Eleanor M Simonsick, Susan M Resnick, Jennifer A Schrack

**Affiliations:** 1 Johns Hopkins Bloomberg School of Public Health, Baltimore, Maryland, United States; 2 National Institute on Aging, Baltimore, Maryland, United States; 3 University of Pennsylvania, Philadelphia, Pennsylvania, United States; 4 Johns Hopkins University and Medical Institutions, Baltimore, Maryland, United States

## Abstract

With aging, brain structural integrity may influence patterns of physical activity (PA) performed in community-dwelling settings. In 281 cognitively-intact adults aged ≥65 years, linear regression models were fitted to examine whether MRI brain volumes (cc), assessed using an automated multi-atlas approach, were cross-sectionally associated with accelerometer-derived: 1) daily PA minutes and 2) activity fragmentation defined as the ratio of # of contiguous PA minutes over total PA minutes x 100. Higher white matter in the parietal and temporal lobes were associated with more daily active minutes (2.8 (SE=1.0) and 3.1 (0.9) min/day, respectively; p<0.005 for both) after adjusting for demographics, behavioral factors, medical conditions, and intracranial volume. Higher white matter in the temporal region was associated with lower fragmentation (-0.15 (0.05) %, p=0.004). Our results suggest sensorimotor-related brain morphometry is connected with both the amount and manner in which PA is performed throughout the day in well-functioning older adults.

